# Systematic Elucidation of the Potential Mechanisms of Core Chinese Materia Medicas in Treating Liver Cancer Based on Network Pharmacology

**DOI:** 10.1155/2020/4763675

**Published:** 2020-04-24

**Authors:** Zhulin Wu, Lina Yang, Li He, Lianan Wang, Lisheng Peng

**Affiliations:** ^1^The Fourth Clinical Medical College of Guangzhou University of Chinese Medicine, Shenzhen 518033, China; ^2^Shenzhen Traditional Chinese Medicine Hospital, Shenzhen 518033, China

## Abstract

**Objective:**

In this study, the data mining method was used to screen the core Chinese materia medicas (CCMMs) against primary liver cancer (PLC), and the potential mechanisms of CCMMs in treating PLC were analyzed based on network pharmacology.

**Methods:**

Traditional Chinese medicine (TCM) prescriptions for treating PLC were obtained from a famous TCM doctor in Shenzhen, China. According to the data mining technique, the TCM Inheritance Support System (TCMISS) was applied to excavate the CCMMs in the prescriptions. Then, bioactive ingredients and corresponding targets of CCMMs were collected using three different TCM online databases, and target genes of PLC were obtained from GeneCards and OMIM. Afterwards, common targets of CCMMs and PLC were screened. Furthermore, a network of CCMMs bioactive ingredients and common target gene was constructed by Cytoscape 3.7.1, and gene ontology (GO) and signaling pathways analyses were performed to explain the mechanism of CCMMs in treating PLC. Besides, protein-protein interaction (PPI) analysis was used to identify key target genes of CCMMs, and the prognostic value of key target genes was verified using survival analysis.

**Results:**

A total of 15 high-frequency Chinese materia medica combinations were found, and CCMMs (including Paeoniae Radix Alba, Radix Bupleuri, Macrocephalae Rhizoma, Coicis Semen, Poria, and Curcumae Radix) were identified by TCMISS. A total of 40 bioactive ingredients (e.g., quercetin, kaempferol, and naringenin) of CCMMs were obtained, and 202 common target genes of CCMMs and PLC were screened. GO analysis indicated that biological processes of CCMMs were mainly involved in response to drug, response to ethanol, etc. Pathway analysis demonstrated that CCMMs exerted its antitumor effects by acting on multiple signaling pathways, including PI3K-Akt, TNF, and MAPK pathways. Also, some key target genes of CCMMs were determined by PPI analysis, and four genes (MAPK3, VEGFA, EGF, and EGFR) were found to be correlated with survival in PLC patients.

**Conclusion:**

Based on data mining and network pharmacology methods, our results showed that the therapeutic effect of CCMMs on PLC may be realized by acting on multitargets and multipathways related to the occurrence and development of PLC.

## 1. Introduction

Primary liver cancer (PLC) is one of the most common and fatal malignant tumors. In 2012, 782,500 new cases of PLC were reported worldwide, being responsible for an estimated 745,000 deaths globally, and PLC ranked as the second leading cause of death from cancer [1, 2]. Moreover, the 5-year survival rate for PLC patients remains low [3]. The most common type of PLC is hepatocellular carcinoma (HCC), which accounts for approximately 70%–85% of all PLC [4]. The risk factors for PLC contain hepatitis C virus (HCV) or hepatitis B virus (HBV) infection, excess alcohol consumption, and aflatoxins. As most patients are diagnosed at an advanced stage and lose the opportunity for surgical resection, the majority of them can only receive palliative treatment. For decades, few treatments have been able to effectively improve the prognosis of advanced PLC, and most of the chemotherapeutic drugs used in advanced PLC have toxic or side effects [5, 6].

Due to the limitations of present therapies, complementary and alternative medicines have been more utilized for the treatment of PLC in the past few decades [7]. In China, the treatment of cancer with traditional Chinese medicine (TCM) has a very long history. TCM doctors are trying to use Chinese materia medica as an adjuvant therapy to ameliorate the quality of life or survival time of patients with PLC. A retrospective study indicated that a combination of Chinese materia medicas and conventional therapies may improve the survival of patients with intermediate or advanced PLC [8]. Several TCM prescriptions have also been confirmed to have anti-PLC effects in basic and clinical research [9–11]. Furthermore, a meta-analysis found that TCM combined with chemotherapy showed significant efficacy and safety in objective response rate, survival time extension, improvement for life quality, and reduction of therapeutic toxicity [7]. Besides, a study reported a rare case of recurrent PLC patient with complete regression of the target lesion with 2 years treatment of Chinese materia medicas [12]. Taken together, as a part of TCM, Chinese materia medicas could effectively improve the quality of life and prolong the survival time in patients with PLC. However, the use of Chinese materia medicas in the treatment of PLC was mostly based on doctors' experience, and it is still unclear which combination of Chinese materia medicas may be effective in treating PLC. In addition, due to the complicated components of Chinese materia medicas, the specific mechanism of Chinese materia medicas in the treatment of PLC is not entirely clear.

At present, the network pharmacology approach can be used to interpret the relationship between diseases, drugs, and targets, showing the network of drug targets from a holistic view [13]. Also, it is of great significance to understand the polypharmacology of drugs and effects of drugs on biological networks using the network pharmacology approach [14]. Additionally, the data mining method has been widely used in TCM, and data mining analysis of prescription is also one of the research hotspots in TCM [15]. Moreover, the data mining method can be utilized to study the rules of Chinese materia medicas in treating diseases and identifying core Chinese materia medicas (CCMMs) from a large number of TCM prescriptions. In this study, the data mining method was used to extract the CCMMs for the treatment of PLC, and we tried to uncover potential mechanisms for CCMMs as a potential therapy for PLC using the network pharmacology approach.

## 2. Materials and Methods

**Figure 1:**
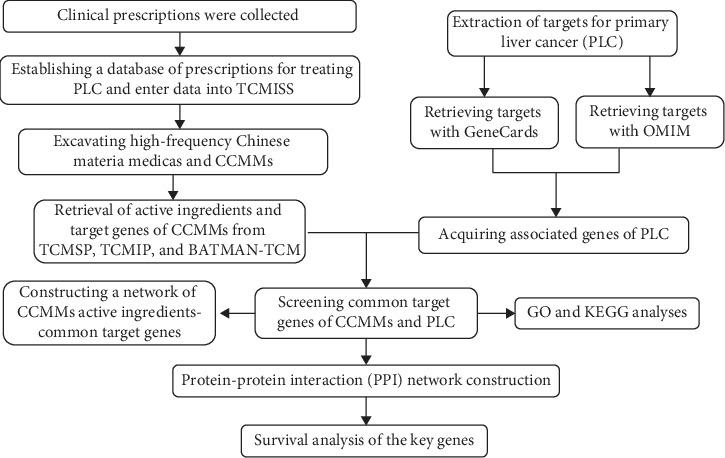
Flow diagram showing the process of the present study.

First of all, TCM prescriptions for the treatment of PLC were collected from the clinical practice of a famous TCM doctor, and then a database of TCM prescriptions for treating PLC was established. Secondly, data mining software named the TCM Inheritance Support System (TCMISS) was used to extract the CCMMs. Subsequently, the ingredients and target genes of the CCMMs were obtained from three different databases. Target genes related to PLC were searched in GeneCards and OMIM databases. In addition, the common target genes of CCMMs and PLC were screened. Furthermore, potential mechanism of CCMMs in treating liver cancer was analyzed, and survival analyses of key target genes were performed. The overall flowchart for this study is displayed in Figure 1.

### 2.1. Prescriptions Data Collation

All TCM prescriptions for the treatment of PLC were acquired from Shenzhen traditional Chinese Medicine Hospital affiliated to Guangzhou University of Chinese Medicine (date: from October 2009 to August 2019), which were prescribed by Professor Chensheng Ouyang, a famous TCM doctor in Shenzhen, China. The inclusion criterion of TCM prescriptions was the first diagnosis was PLC, and exclusion criteria were (1) duplicate prescriptions of the same patients; (2) prescriptions were primarily for treating acute symptoms such as cold, cough, diarrhea, and vomiting; and (3) external prescriptions. Based on data mining methods, TCMISS was applied to extract the high-frequency Chinese materia medicas and CCMMs of the prescriptions. TCMISS is software widely used in TCM prescription data analyses, which integrates general statistics, text mining, association rules, and complex system entropy clustering methods [15, 16]. Through the “prescription management” module in the “platform management system” of TCMISS, the prescriptions data were entered. To ensure the reliability and accuracy of the results, two authors (Yang and He) examined the data after completion of the data entry.

### 2.2. Data Mining of Prescriptions and Extraction of CCMMs

The association rules method in TCMISS was used to identify the high-frequency combinations of Chinese materia medicas, and then CCMMs were obtained. Specifically, the “prescription analysis” module of “data analysis” section in TCMISS was selected to identify the frequency and the combinations of Chinese materia medicas. Analysis of prescription rules was carried out under the condition that support the degree was 200 and the confidence score was greater than or equal to 0.95.

### 2.3. Bioactive Ingredients and Targets of CCMMs

TCM Systems Pharmacology Database and Analysis Platform (TCMSP, http://tcmspw.com/tcmsp.php) [17], Integrative Pharmacology-based Research Platform of TCM (TCMIP, http://www.tcmip.cn/) [18], and Bioinformatics Analysis Tool for Molecular Mechanism of TCM (BATMAN-TCM, http://bionet.ncpsb.org/batman-tcm/) [19], were utilized to retrieve the ingredients of CCMMs. Each ingredient was annotated using the Mol ID from TCMSP, and the parameters for screening bioactive ingredients were set as follows: drug-likeness (DL) ≥ 0.18 and oral bioavailability (OB) ≥ 30% [20]. Besides, targets related to bioactive ingredients were obtained by TCMSP. All target names were converted into corresponding gene names using UniProt (https://www.uniprot.org/).

### 2.4. Prediction of Target Genes of PLC

The databases “GeneCards” (https://www.genecards.org/) [21] and “Online Mendelian Inheritance in Man” (OMIM, https://omim.org/) [22] were searched using keywords primary liver cancer, hepatocellular carcinoma; HCC; hepatoma; hepatic cancer; and hepatic carcinoma. Potential therapeutic target genes of PLC were gained after summarizing and eliminating duplication.

### 2.5. The Network of CCMMs Bioactive Ingredients and Common Target Genes

Common target genes of CCMMs and PLC were screened using the Draw Venn Diagram online tool (http://bioinformatics.psb.ugent.be/webtools/Venn/). In order to fully understand the molecular mechanism, the network of CCMMs bioactive ingredients and common targets was constructed by using Cytoscape 3.7.1.

### 2.6. Gene Ontology (GO) and Signaling Pathway Analyses

GO and Kyoto Encyclopedia of Genes and Genomics (KEGG) signaling pathway analyses for common target genes were performed by the Database for Annotation, Visualization, and Integrated Discovery (DAVID, https://david.ncifcrf.gov/) [23]. In addition, GO enrichment analysis was performed according to three types, including GO biological processes (GO-BP), GO cellular component (GO-CC), and GO molecular function (GO-MF). The results of GO analysis were presented by a barplot using “ggplot2” package in *R* software (version 3.6.0). Furthermore, top 20 key KEGG pathways were identified according to the order of gene count. In the light of the biological functions of common targets and correlative signaling pathways, the mechanism of CCMMs against PLC was discussed. In GO and KEGG analyses, a false discovery rate (FDR) below 0.05 was considered significant.

### 2.7. Protein-Protein Interaction (PPI) Analysis and Screening for Key Genes

The common target genes were input into Search Tool for the Retrieval of Interacting Genes (STRING) database (https://string-db.org/) [24] to construct a protein-protein interaction (PPI) network by setting the minimum required interaction score at 0.400. Furthermore, CytoNCA plugin [25] in Cytoscape 3.7.1 was used to perform a topological analysis, including degree centrality (DC), betweenness centrality (BC), and closeness centrality (CC), for key genes screening. The core PPI network was presented by using NetworkAnalyzer plugin in Cytoscape.

### 2.8. Correlation between Key Genes and Prognosis

After obtaining the key target genes from the PPI network, we used the Kaplan–Meier Plotter (http://kmplot.com/analysis/) to explore the association between key genes expression and prognosis in patients with PLC. The Kaplan–Meier Plotter includes data on survival prognosis of liver cancer in The Cancer Genome Atlas (TCGA) database [26]. In the present study, survival analysis was performed for key genes with DC > 90, and overall survival (OS) was analyzed by the Kaplan–Meier (K–M) survival analysis (log-rank test). A *p* value less than 0.05 was considered significant.

## 3. Results

### 3.1. Results of Data Mining by TCMISS

**Figure 2:**
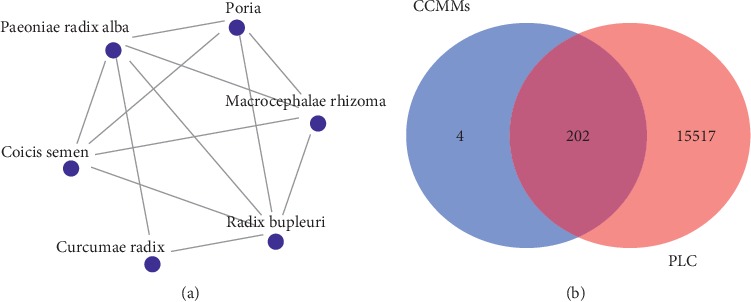
Network of CCMMs by TCMISS (a) and Venn diagram for common target genes of CCMMs and PLC (b).

A total of 326 TCM prescriptions for the treatment of PLC were collected. The most frequently used Chinese materia medicas were Paeoniae Radix Alba, Radix Bupleuri, Macrocephalae Rhizoma, Coicis Semen, Poria, Curcumae Radix, Dioscoreae Rhizoma, and curcumae rhizome. Top 20 frequently-used Chinese materia medicas were shown in Table 1. In addition, 15 most commonly used combinations of Chinese materia medicas were acquired, including 6 kinds of Chinese materia medicas, and the top 15 combinations of Chinese materia medicas are displayed in Table 2. Moreover, TCMISS was utilized to demonstrate the application mode of Chinese materia medicas in a networked view, and the Chinese materia medicas shown in Figure 2(a) were CCMMs.

### 3.2. Results of Bioactive Ingredients and Targets of CCMMs

The data of CCMMs ingredients from three different databases are listed in Table 3. Also, bioactive ingredient count of each core Chinese materia medica is presented in Table 3. Following removal of the duplicates, a total of 40 ingredients were identified, and 206 potential target genes of CCMMs were obtained.

### 3.3. Potential Target Genes of PLC

Fifteen thousand four hundred and seventy-eight and three hundred and thirty-eight PLC-related target genes were extracted from GeneCards and OMIM databases, respectively. A total of 15719 therapeutic target genes for PLC were obtained after the elimination of duplicates.

### 3.4. Construction of CCMMs Bioactive Ingredients-Common Target Genes Network

**Figure 3:**
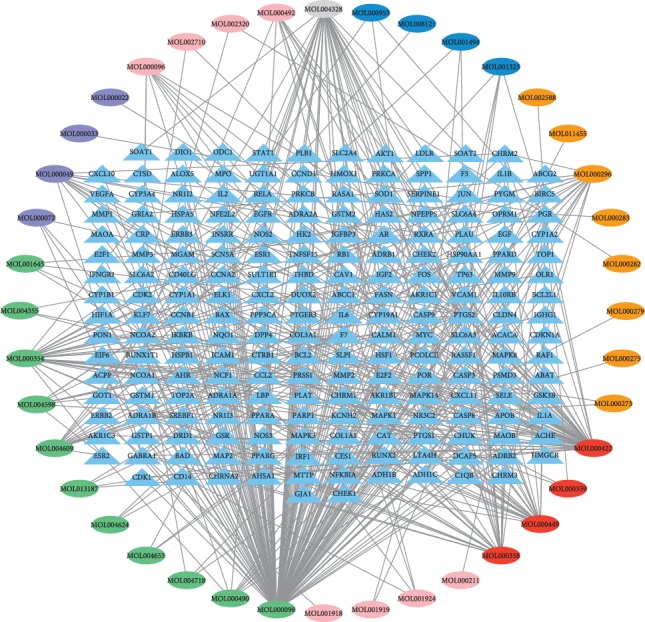
The network of CCMMs and common target genes. Light blue triangles represent the common target genes of PLC and CCMMs bioactive ingredients. The ovals indicate the bioactive ingredients of CCMMs, in which red represents the common bioactive ingredients of multiple Chinese materia medicas, and pink, purple, green, orange, blue, and gray represent the bioactive ingredients of Paeoniae Radix Alba, Macrocephalae Rhizoma, Radix Bupleuri, Poria, Coicis Semen, and Curcumae Radix, respectively.

A total of 202 common targets of CCMMs and PLC were identified by Draw Venn Diagram online tool (Figure 2(b)). A network of CCMMs bioactive ingredients-common target genes including 242 nodes (202 target genes, 40 bioactive ingredients) and 447 edges (interactions) was established (Figure 3), and the details of bioactive ingredients are shown in Table 4.

### 3.5. Results of GO and KEGG Signaling Pathway Analyses

**Figure 4:**
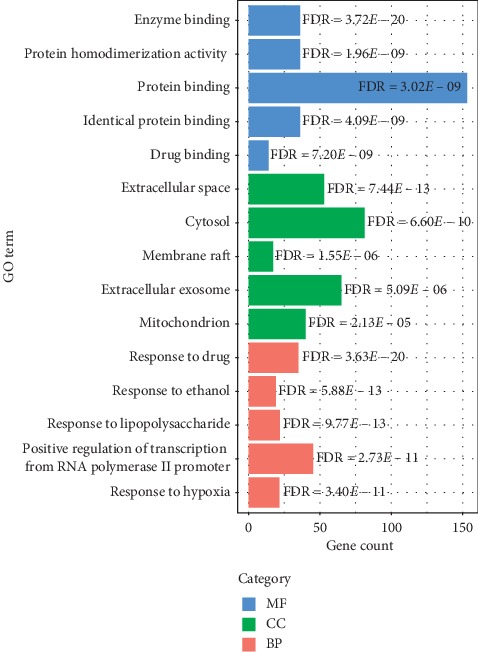
The barplot for GO analysis of common target genes. The top five significantly enriched GO terms in each category ranked according to FDR. FDR: false discovery rate.

The top 5 enrichment results in each category of GO analysis are displayed in Figure 4 (FDR < 0.05). The GO-BP involved 74 enrichment results, including response to drug and response to ethanol, and 23 enrichment results were related to GO-MF which cover the enzyme binding, protein homodimerization activity, etc. In addition, there were 14 enrichment results in GO-CC, including extracellular space and cytosol. Moreover, the results of the top 20 KEGG signaling pathways are listed in Table 4. According to the results of KEGG, the common target genes of PLC and CCMMs were mainly enriched in cancer-related signaling pathways, such as pathways in cancer, PI3K-Akt pathway, TNF pathway, and MAPK pathway (Table 5).

### 3.6. Results of PPI Network Analysis

**Figure 5:**
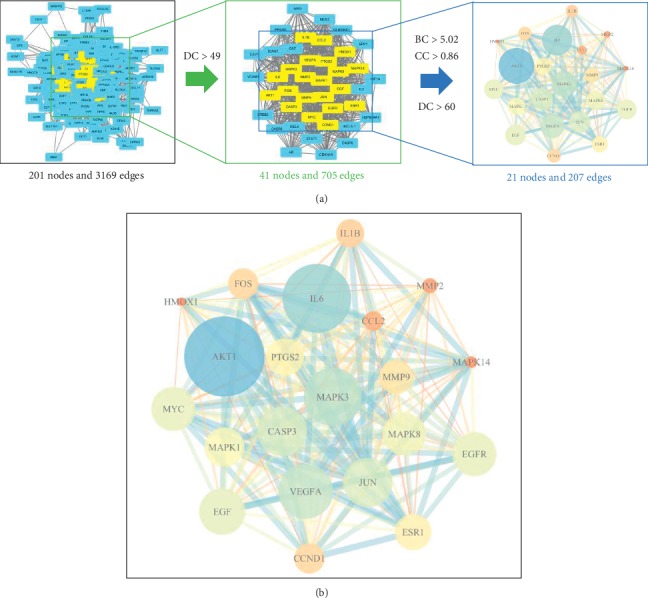
PPI networks of CCMMs and PLC. (a) The screening process of the target genes. The screening criteria by which the key target genes were identified were DC > 49, BC > 5.02, and CC > 0.86. (b) PPI network of CCMMs and PLC with 21 nodes and 207 edges. Nodes indicate key target genes. The size of the nodes and edges corresponds to the value of degree and combine score, respectively. The color of the nodes represents the value of degree. The darker (blue) the color, the higher the degree. DC: degree centrality; BC: betweenness centrality; CC: closeness centrality.

The rough PPI network with 201 nodes and 3169 edges was constructed using STRING. Because nodes with DC only more than twofold median DC of all nodes can be significant targets in the PPI network [27], we used twice the median of the DC value to generate a subnetwork. After that, target genes with BC ≥ median and CC ≥ median were selected to construct the core network and obtain key nodes (genes). The screening process is shown in Figure 5(a), and the key genes in the core network is presented in Figure 5(b). The results of PPI analysis demonstrated that AKT1, IL6, MAPK3, VEGFA, and CASP3 could be considered as key target genes of CCMMs in the treatment of PLC.

### 3.7. Effect of Key Genes on OS of PLC Patients

**Figure 6:**
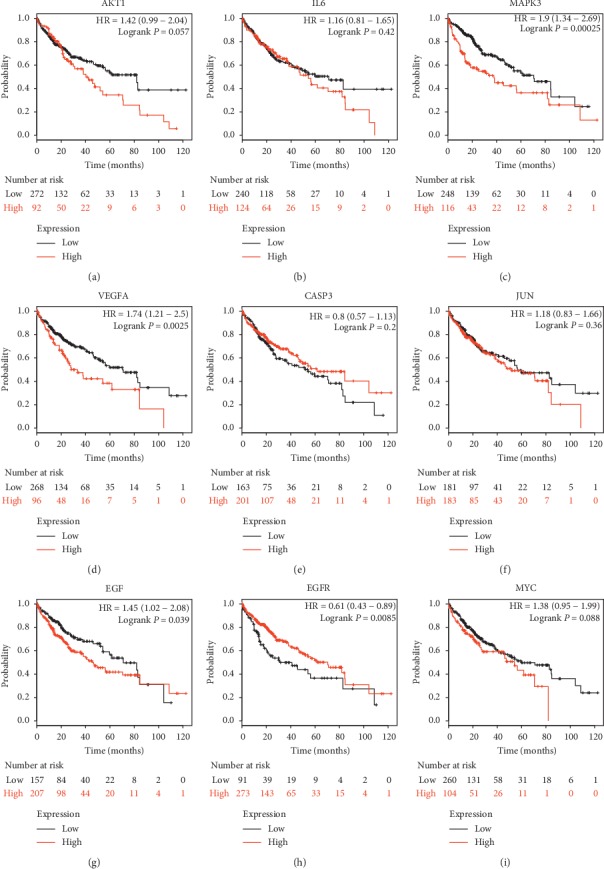
The prognostic values of expression levels of key genes in PLC patients. (a–i) The K–M survival curves of nine top key genes. High expression levels of MAPK3 (c), VEGFA (d), EGF (g) were associated with worse OS in PLC patients, and high expression level of EGFR (h) was correlated with longer OS. (a, b, e, f, i) Other genes showed no significant difference. OS: overall survival; K–M: Kaplan–Meier.

The associations between nine key genes (AKT1, IL6, MAPK3, VEGFA, CASP3, JUN, EGF, EGFR, and MYC) with the DC > 90 and OS rate were analyzed by the K–M method. Survival curves of the key genes are shown in Figures 6(a)–6(i). Our data demonstrated that high expressions of MAPK3, VEGFA, and EGF were significantly related to poor prognosis (Figures 6(c), 6(d), 6(h)), and high expression of EGFR was correlated with better prognosis (Figure 6(h)). All other key genes showed no significant difference, Figures 6(a)–6(i).

## 4. Discussion

The results of data mining demonstrated that CCMMs for PLC comprised six Chinese materia medicas, including Radix Alba, Radix Bupleuri, Macrocephalae Rhizoma, Coicis Semen, Poria, and Curcumae Radix. According to TCM theories, these CCMMs can be applied to disperse stagnated liver Qi, reinforce Qi, and nourish Xue (blood). In TCM, the pathogenesis of PLC is usually thought to be caused by liver Qi stagnation and deficiency of qi and Xue. Thus, data mining results are in line with the theories of TCM. Furthermore, the results of network pharmacology revealed that the bioactive ingredients of CCMMs, including quercetin, kaempferol, naringenin, isorhamnetin, beta-sitosterol, and stigmasterol were related to more target genes, which were the key bioactive ingredients. Quercetin has been proved to induce apoptosis of liver cancer cells (SMMC-7721), and its mechanism could be related to the inhibition of activation of Akt by PTEN gene overexpression [28]. Kaempferol is one of the general flavonoids and has been reported to curb proliferation and migration of liver cancer HepG2 cells through inactivating the PI3K/Akt signaling pathway [29]. Recent research indicated that naringenin could induce apoptosis and regulate the MAPK pathway so as to prevent the occurrence of liver cancer [30]. Choi [31] reported that isorhamnetin could inhibit the proliferation of human hepatocarcinoma Hep3B cells by arresting the cell cycle at the G2/M phase. Zhang [32] found that beta-sitosterol may inhibit proliferation and induced apoptosis of HepG2 cells through membrane death receptor and the mitochondrial pathway. In addition, a study showed that stigmasterol could induce apoptosis of HepG2 cells by upregulating proapoptotic genes (Bax, p53) and downregulating antiapoptotic gene (Bcl-2) expressions [33]. The interaction network of CCMMs bioactive and common targets revealed that CCMMs are able to serve an antitumor role through multiple bioactive ingredients and targets, which is very consistent with the holism and the thought of treatment based on syndrome differentiation in TCM.

In the present study, GO and KEGG analyses were used to predict the underlying mechanism of CCMMs in treating PLC. The result of GO analysis indicated that common targets were significantly enriched in the mitochondrion, extracellular exosome, membrane raft, cytosol, and extracellular space, showing that these genes are involved in various cell metabolisms. As for GO-MF, the genes were involved in drug binding, identical protein binding, protein binding, protein homodimerization activity, and enzyme binding, which are correlated with the liver metabolic function. In GO-BP, common target genes were most enriched in response to hypoxia, positive regulation of transcription from RNA polymerase II promoter, response to lipopolysaccharide, response to ethanol, and response to drug, suggesting that CCMMs may be associated with cell apoptosis, liver metabolism, immune system, and drug metabolism. KEGG analysis showed that CCMMs may produce anti-PLC effects via multiple pathways, including pathways in cancer, hepatitis B, PI3K-Akt, TNF, MAPK, hepatitis C, HIF-1, toll-like receptor, and Ras signaling pathway. Previous studies have reported that the expression of Akt in PLC cells is significantly higher than that in normal liver tissues [34], and Chinese materia medicas could regulate the PI3K/Akt/mTOR signaling pathway to induce cell apoptosis and inhibit the growth of hepatoma cells [35]. Hence, the PI3K/Akt pathway may provide novel target drugs for HCC treatment. In addition, the literature has shown that there was a positive correlation between HBV infection and PLC, and HBV that integrate in the host genome can lead to the inactivation of tumor suppressor genes or the activation of protooncogenes [36–38]. It has been reported that TNF serves a critical role in the development of liver cancer [39]. Also, HIF-1 plays an important role in immune escape and epithelial-mesenchymal transformation of PLC [40], which shows great prospects in the treatment of PLC. Furthermore, research indicated that both HBV and HCV-related hepatocarcinogenesis activate the Ras/MAPK pathway which is associated with a poor prognosis [41–43]. Additionally, HCV-induced EGFR-ERK signaling may promote the development and progression of PLC [44]. The toll-like receptor (TLR) pathway is involved in the initiation, progression, and metastasis of liver cancer, which may become a new target for targeted therapies of PLC [45]. Patients with PLC are susceptible to infections due to hypoimmunity, which may be related to tuberculosis, HTLV-I infection, influenza A, and Chagas disease pathways. Besides, other signaling pathways in this study are also associated with cancer. These findings indicated that CCMMs could be used in treating PLC by regulating multiple pathways related to the occurrence and development of cancer.

Several key target genes of CCMMs were identified by PPI network construction, and four of the top nine genes were found to be correlated with OS in PLC patients. An experimental study showed that overexpression of AKT1 may promote the migration and invasion of PLC cells (HepG2) [46]. Inflammatory plays a key role in the occurrence, development, and metastasis of PLC. IL-6 is highly upregulated in PLC, which is correlated with rapid progression from hepatitis to PLC [47]. Previous studies found that CASP3 (Caspase-3) can prevent chemical-induced PLC by suppressing p38 activation and hepatocyte death [48], and total flavonoids from Ampelopsis grossedentata (Teng cha) exerts an antitumor effect on PLC by upregulating the expression of CASP3 [49]. It is reported that PLC patients with high phosphorylated MAPK3 (also called ERK1) had significantly higher cancer recurrence and worse OS [50], and the impact of MAPK3 on survival was also verified by the survival curve in this study. Angiogenesis is considered as a key process in the development of cancers. VEGFA is an angiogenesis inducer in PLC, and expression of VEGFA in PLC was significantly higher than that in adjacent normal tissues [51]. The survival analysis demonstrated that PLC patients with high expression of VEGFA had a poor prognosis, suggesting its important role in PLC therapy. EGF, being a growth factor, plays a critical role in cell proliferation, survival, and migration by binding to its receptor EGFR. Evidence showed that high expression of EGF can induce highly malignant PLC, and activated EGF/EGFR signaling is closely related to intrahepatic metastasis [52]. Previous studies suggested that EGFR is overexpressed or mutated in PLC cells and may be closely related to the formation, invasive growth, and clinical characteristics of PLC [53, 54]. However, our data showed that high expression of EGFR is correlated with better prognosis, and further research is required to study the mechanism of this association. Last but not least, JUN and MYC are also involved in the initiation and progression of HBV-associated PLC [55, 56]. In general, these genes are involved in the inflammatory response, tumor neovascularization, and the occurrence and development of PLC, and some of these genes are associated with the prognosis of PLC.

In our study, the CCMMs for the treatment of PLC were identified from a large number of clinical prescriptions using the data mining approach, which may provide a useful reference for the clinical practice of TCM. Furthermore, the network pharmacology method was applied to acquire potential targets and signaling pathways of CCMMs in the treatment of PLC. According to the results of network pharmacology, CCMMs may exert antitumor effects on PLC by inhibiting the proliferation, invasion, and metastasis of PLC cells and by inducing cell apoptosis in PLC. Moreover, some of the pathways (e.g., PI3K-Akt, Ras/MAPK, HIF-1, TNF, and toll-like receptor pathway) and target genes (e.g., AKT1, IL6, MAPK3, EGF, and EGFR) may provide a novel approach for studying the mechanism of Chinese materia medicas in treating PLC. In the present study, the K–M survival analysis was used to verify the prognostic value of key genes in PPI, and four key genes of CCMMs were found to be significantly related to OS in patients with PLC, which made the results of network pharmacology more reliable. However, the results in this paper depend on the reported ingredients and targets of CCMMs. With the development of experimental technology, more and more molecular targets for PLC therapy as well as new ingredients of Chinese materia medicas will be discovered, which may be helpful to enrich the results of this paper. Besides, future experiments are required to validate the findings of the present study.

## 5. Conclusions

In conclusion, based on the combination of data mining and network pharmacology, we predicted that the therapeutic effect of CCMMs on PLC may be realized by acting on multitargets and multipathways related to the occurrence and development of PLC, which laid a foundation for further experimental study.

## Figures and Tables

**Table 1:** Top 20 Chinese materia medicas in all TCM prescriptions.

Number	Chinese materia medicas	Freq	Number	Chinese materia medicas	Freq
1	Paeoniae radix alba	301	11	Amomi fructus	123
2	Radix bupleuri	292	12	Codonopsis radix	116
3	Macrocephalae rhizoma	279	13	Eupolyphaga	114
4	Coicis semen	278	14	Scutellariae radix	104
5	Poria	253	15	Semen dolichoris album	95
6	Curcumae radix	224	16	Citrus reticulata blanco	89
7	Dioscoreae rhizoma	172	17	Fructus gardeniae	76
8	Curcumae rhizoma	166	18	Amomi fructus rotundus	72
9	Trionycis carapax	160	19	Astmgali radix	70
10	Fructus aurantii	127	20	Moutan cortex	69

**Table 2:** Top 15 commonly used combinations of Chinese materia medicas.

Number	Most commonly used combinations of Chinese materia medicas	Freq
1	Paeoniae radix alba, radix bupleuri	289
2	Paeoniae radix alba, coicis semen	263
3	Radix bupleuri, coicis semen	256
4	Macrocephalae rhizoma, paeoniae radix alba	255
5	Paeoniae radix alba, radix bupleuri, coicis semen	254
6	Macrocephalae rhizoma, radix bupleuri	246
7	Macrocephalae rhizoma, paeoniae radix alba, radix bupleuri	244
8	Macrocephalae rhizoma, coicis semen	243
9	Macrocephalae rhizoma, poria	236
10	Paeoniae radix alba, poria	231
11	Macrocephalae rhizoma, paeoniae radix alba, coicis semen	228
12	Macrocephalae rhizoma, radix bupleuri, coicis semen	222
13	Curcumae radix, paeoniae radix alba	221
14	Curcumae radix, radix bupleuri	220
15	Radix bupleuri, poria	220

**Table 3:** The data of CCMMs ingredients from three different databases.

CCMMs	TCMSP	TCMIP	BATMAN	Bioactive ingredient counts
Paeoniae radix alba	85	54	35	18
Radix bupleuri	349	62	82	18
Macrocephalae rhizoma	55	20	11	7
Coicis semen	38	2	3	9
Poria	34	33	21	18
Curcumae radix	222	11	27	15

**Table 4:** The information of screened bioactive ingredients in CCMMs.

Mol ID	Bioactive ingredients	Gene count	OB	DL
MOL000098	Quercetin	140	46.43	0.28
MOL000422	Kaempferol	55	41.88	0.24
MOL004328	Naringenin	34	59.29	0.21
MOL000354	Isorhamnetin	30	49.6	0.31
MOL000358	Beta-sitosterol	28	36.91	0.75
MOL000449	Stigmasterol	27	43.83	0.76
MOL000296	Hederagenin	17	36.91	0.75
MOL000049	3*β*-Acetoxyatractylone	14	54.07	0.22
MOL004609	Areapillin	14	48.96	0.41
MOL004598	3,5,6,7-Tetramethoxy-2-(3,4,5-trimethoxyphenyl)chromone	10	31.97	0.59
MOL000096	(−)-Catechin	9	49.68	0.24
MOL000492	(+)-Catechin	9	54.82	0.24
MOL000490	Petunidin	8	30.05	0.31
MOL001323	Sitosterol alpha1	5	43.28	0.78
MOL000072	8*β*-Ethoxy atractylenolide III	4	35.05	0.21
MOL001645	Linoleyl acetate	4	42.1	0.2
MOL001924	Paeoniflorin	4	53.87	0.79
MOL004653	(+)-Anomalin	4	46.06	0.66
MOL013187	Cubebin	4	57.13	0.64
MOL000359	Sitosterol	3	36.91	0.75
MOL000953	CLR	3	37.87	0.68
MOL001494	Mandenol	3	42	0.19
MOL004355	Spinasterol	3	46.43	0.28
MOL004624	Longikaurin A	3	47.72	0.53
MOL004718	*α*-Spinasterol	3	42.98	0.76
MOL000273	(2R)-2-((3S,5R,10S,13R,14R,16R,17R)-3,16-dihydroxy-4,4,10,13,14-pentamethyl-2,3,5,6,12,15,16,17-octahydro-1H-cyclopenta(a)phenanthren-17-yl)-6-methylhept-5-enoic acid	2	30.93	0.81
MOL001919	Palbinone	2	43.56	0.53
MOL002320	Poriferast-5-en-3beta-ol	2	54.83	0.24
MOL002710	Pyrethrin II	2	54.83	0.24
MOL000022	14-acetyl-12-senecioyl-2E,8Z,10E-atractylentriol	1	63.37	0.3
MOL000033	(3S,8S,9S,10R,13R,14S,17R)-10,13-dimethyl-17-((2R,5S)-5-propan-2-yloctan-2-yl)-2,3,4,7,8,9,11,12,14,15,16,17-dodecahydro-1H-cyclopenta(a)phenanthren-3-ol	1	36.23	0.78
MOL000211	Mairin	1	55.38	0.78
MOL000275	Trametenolic acid	1	38.71	0.8
MOL000279	Cerevisterol	1	37.96	0.77
MOL000282	Ergosta-7,22E-dien-3beta-ol	1	43.51	0.72
MOL000283	Ergosterol peroxide	1	40.36	0.81
MOL001918	Paeoniflorgenone	1	87.59	0.37
MOL002588	Eburicol	1	44.17	0.82
MOL008121	2-Monoolein	1	34.23	0.29
MOL011455	20-Hexadecanoylingenol	1	44.17	0.83

**Table 5:** Results of KEGG pathway enrichment (top 20).

Name of pathways	Gene numbers	FDR
Hsa05200: pathways in cancer	50	2.62*E* − 18
Hsa05161: hepatitis B	32	2.03*E* − 17
Hsa04151: PI3K-Akt signaling pathway	30	1.86*E* − 05
Hsa05152: tuberculosis	25	2.35*E* − 08
Hsa05215: prostate cancer	24	2.09*E* − 14
Hsa04668: TNF signaling pathway	24	2.42*E* − 12
Hsa04010: MAPK signaling pathway	24	1.66*E* − 04
Hsa05166: HTLV-I infection	24	1.78*E* − 04
Hsa05164: influenza A	23	6.95*E* − 07
Hsa05205: proteoglycans in cancer	23	1.00*E* − 05
Hsa05206: micro-RNAs in cancer	23	0.005433
Hsa05145: toxoplasmosis	22	5.30*E* − 10
Hsa05160: hepatitis C	21	1.92*E* − 07
Hsa04510: focal adhesion	21	4.05*E* − 04
Hsa05212: pancreatic cancer	20	1.71*E* − 12
Hsa04066: HIF-1 signaling pathway	20	3.78*E* − 09
Hsa05142: Chagas disease (American trypanosomiasis)	20	1.69*E* − 08
Hsa04620: toll-like receptor signaling pathway	20	2.40*E* − 08
Hsa04024: cAMP signaling pathway	19	0.004254
Hsa04014: Ras signaling pathway	19	0.026935

## Data Availability

The data that support the findings of this study are openly available in http://tcmspw.com/tcmsp.php, http://www.tcmip.cn/, http://bionet.ncpsb.org/batman-tcm/, https://www.uniprot.org/, https://www.genecards.org/, https://omim.org/, http://bioinformatics.psb.ugent.be/webtools/Venn/, https://string-db.org/, https://david.ncifcrf.gov/, and http://kmplot.com/analysis/.
